# Perceptions and Experiences of Caregiver-Employees, Employers, and Health Care Professionals With Caregiver-Friendly Workplace Policy in Hong Kong: Thematic Analysis

**DOI:** 10.2196/58528

**Published:** 2025-02-10

**Authors:** Maggie Man-Sin Lee, Eng-kiong Yeoh, Eliza Lai-Yi Wong, Xue Bai, Nelson Chun-Yiu Yeung, Catherine French, Henock Taddese

**Affiliations:** 1 The Jockey Club School of Public Health and Primary Care Chinese University of Hong Kong Hong Kong China (Hong Kong); 2 Department of Applied Social Science The Hong Kong Polytechnic University Hong Kong China (Hong Kong); 3 Department of Health and Life Sciences King's College London London United Kingdom; 4 School of Public Health Faculty of Medicine, Imperial College London London United Kingdom

**Keywords:** caregiver employees, workplace, discrimination, dual roles, caregiver burden

## Abstract

**Background:**

Caregiver-employees (CEs) for older adults experience a high burden to fulfill their dual roles. Caregiver-friendly workplace policy (CFWP) has been used in many countries to balance employment and caregiving duties, but it is a relatively new concept in Hong Kong.

**Objective:**

This study explored the views and experiences of CEs, employers, and health care professionals regarding CFWP (specifically for older adult caregivers) in Hong Kong.

**Methods:**

This study explored the CFWP-related views and experiences in Hong Kong using 15 in-depth interviews with purposively sampled CEs for older adults, employers, and health care professionals.

**Results:**

Two context-related themes (“lacking leadership” and “unfavorable culture”) were identified with thematic analysis. They explain the absence of CFWP in Hong Kong due to the lack of governmental and organizational leadership, and the additional burden experienced by CEs because of the working culture that underpins work-life separation, overprizing business interest, and unsympathetic corporate attitude. Implicit voice theory was applicable in explaining CEs’ nondisclosure about their status at work due to potential risks. In addition, the two facilitation-related themes (“role struggle” and “inadequate support”) identified in this study exhibit how the dual role had positive and negative spillover effects on each other and the inadequacy of social welfare and health care support systems.

**Conclusions:**

We strongly recommend exploring and adopting potential CFWP in Hong Kong, considering the complexity of factors identified in this study.

## Introduction

### Background

In 2020, one billion people globally were aged 60 years or older, which is expected to double by 2050 [1]. This unprecedented increase in the aging population poses numerous economic, political, social, and health care challenges [2]. One of the pressing issues has been providing daily care to older adults. However, the size of the professional and trained workforce has not increased proportionately to meet this demand [3], driving innovations in other fields, such as robotics, to relieve some of the unmet demands of older people care [4]. Even in countries where welfare systems are well established, family caregivers provide most of the care and support for older adults [5].

Consequently, caregivers experience high burdens, strain, and poor mental health outcomes such as burnout, anxiety, and depression, as noted by several studies [6,7]. Many caregivers may simultaneously engage in paid employment, referred to as caregiver-employees (CEs), who may experience additional burdens due to high professional and caregiving demands [8]. CEs experience significant impairments [7]; they are three times more vulnerable to adverse health issues than non-CEs [9]. The progressive decline in care recipients’ functional capacity requires higher physical effort and caregiving time, resulting in poor health and depression among CEs [10].

Health care professionals (HPs) have traditionally provided psycho-educational support to promote caregivers’ competency and well-being [11]. In addition, employers (ERs) are increasingly adopting caregiver-friendly workplace policies (CFWPs) to mitigate some of the caregivers’ burden [7]. For instance, about 80% of ERs in the United States provide some CFWP [12]. CFWP typically includes flexible working arrangements, support services, and paid or unpaid leave [13] to help CEs manage their multiple roles and improve their work-life balance [7].

The growing international commitment to sustainable organizational behavior in which employee well-being is a significant determinant of their productivity, and therefore, organizational performance has accelerated the development and widespread adoption of CFWP [14-16]. For instance, studies have shown that in addition to employee well-being, CFWP may be critical for promoting the productivity of CEs [17] as they have been associated with improved overall health of CEs by reducing occupational and overall stress, minimizing work interruptions, and improving performance [18]. There are also direct economic benefits accruing from adopting CFWP. For example, educating CEs about their caregiving activities generates a net benefit ranging from US $48,010 to US $675,657 for CEs and ERs [19].

However, the three core stakeholders of CFWP may have different perspectives on the objectives, gaps, and limitations of the existing policies against the context of, for example, personal needs (for CEs), imparting caregiving competence (for HPs), or improving organizational performance (for ERs). Thus, exploring CFWP-related experiences and perceptions of CEs’ and other stakeholders, especially HPs and ERs, is imperative. While several studies have reported the types and impact of CFWP [13,17], there is a paucity of studies reporting stakeholders’ perspectives on CFWPs. Although we could not identify any study exploring the perspectives of CEs, one study explored the perspectives of managers working in the Canadian health care sector [20]. In addition, we could not identify any academic literature, public policy, or articulated organizational policy specifically devised for CEs caring for older adults.

Therefore, this study explored the CFWP-related views and experiences of CEs from Hong Kong caring for older adults, along with the views and experiences of local HPs and ERs with prior experience with formulating or implementing CFWP.

### Conceptual Framework

Since CFWP is a relatively new idea in Hong Kong, we adopted the Promoting Action on Research Implementation in Health Services (PARIHS) framework for this explorative qualitative study. PARIHS was developed by the Royal College of Nursing in the United Kingdom as a conceptual framework of interacting elements to implement evidence-based practices [21]. The framework’s key constructs, namely evidence, context, and facilitation, were used to guide the interview with participants regarding any future policy implementation.

**Figure 1:**
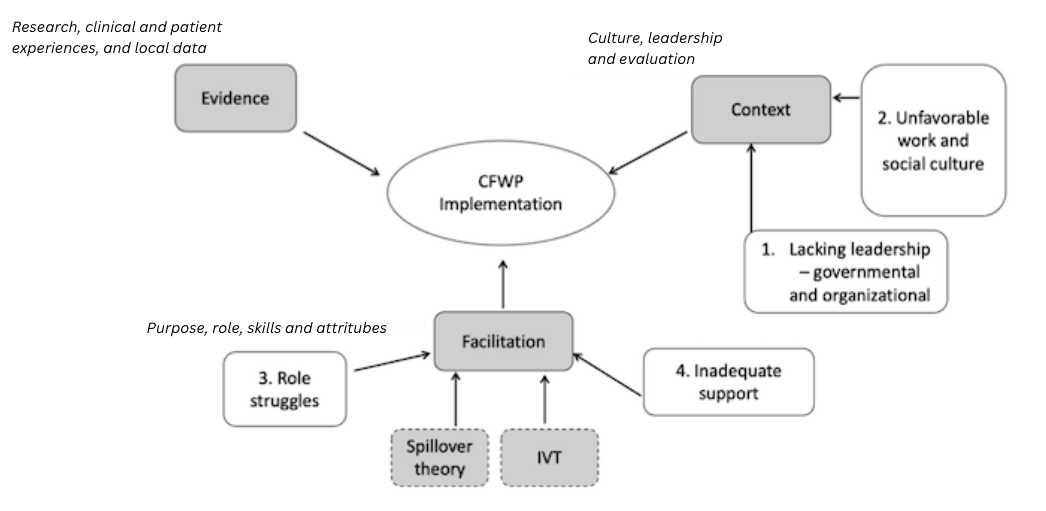
Schematic diagram outlining the conceptual framework of the study. CFWP: caregiver-friendly workplace policy; IVT: implicit voice theories.

To further add to the framework and explicate the facilitating factors, spillover theory [22] and implicit voice theory (IVT) [23] were used. The two theories orientate the questions from the point of view of the CEs, whereby spillover theory explores whether the interface between the microsystems of work and family is positive or negative [24], and IVT takes the gaze toward any perceived risks of inappropriateness of speaking up or disclosure of status in an organizational hierarchy [25]. These theories are fundamental in exploring the facilitation of CFWP, in other words, the expectation of and potential actions of stakeholders vis-a-vis the relatively new policy idea. Spillover theory aids the exploration of the interactions of the dual roles—work and caring—while IVT sheds light on whether CEs could or would disclose their CE status and advance their interests. Subsequently, the guiding theoretical framework adapted for this study effectively superimposes IVT and spillover theories on top of the PARIHS framework (Figure 1).

## Methods

### Study Setting

The economic and sociocultural factors in Hong Kong put carers in a particularly precarious position. Economically, laissez-faire has long been the foundation of Hong Kong’s stability and prosperity [26,27]. However, free-market neoliberalism has failed Hong Kong in terms of increasing social inequality [28,29], leaving the economically and socially vulnerable even more susceptible to discrimination and unfair treatment. In our case, the CEs care for sick older people without their dual role being recognized in the workplace. Socioculturally, the experience of CEs is complicated by filial piety, the social value of reverence, which significantly constrains the attitudes and behaviors of the caregivers since Hong Kong households emphasize moral obligation in a strictly hierarchical sense based on the recognition of aid and care given to older adults [30].

The salient economic and sociocultural context of Hong Kong is further complicated by Hong Kong’s aging population, which is expected to rise sharply from 17% in 2016 to 37% by 2066 [31]. Similarly, the older people dependency ratio is expected to increase from 5:1 in 2015 to 1.8:1 by 2064 [32]. The older people dependency ratio is attributed to the high prevalence of chronic disease in the older population; about 65% of the older adults in Hong Kong have a chronic condition, while one-third have at least two chronic conditions [33]. Furthermore, Hong Kong has the fourth highest cost of living among cities globally [34]. As a result, about 59.6% of the Hong Kong population is significantly engaged in the workforce [35]. These population trends indicate a high need for CFWP in Hong Kong and underscores the urgent need to scale up CFWP in Hong Kong. This need also emerged in our recent study in which 7% of CEs in Hong Kong caring for people with Alzheimer disease had signs of probable clinical depression and 10% possible mild depression [36]. Furthermore, the responses of this study’s participants indicated that CFWPs can directly improve their mental well-being and organizational performance [36].

### Study Design

This study was envisioned as theory-led research, considering both conventional theories in the field of CEs study and additional selected theories fitting into the context. Spillover theory and IVT were selected to guide and inform this study’s design, perspectives, and interpretative lens.

Qualitative case studies are particularly appropriate when contextual conditions believed to be relevant to the phenomenon are under investigation and when boundaries between the phenomenon and context are unclear [37]. Given the absence of CFWP in Hong Kong, the contextual conditions the CEs are subjected to are relevant to CFWP because the potential construction of CFWP is firmly based on the context at hand. Therefore, a qualitative case study methodology involving the three core stakeholders of CFWP, that is, CEs, HPs, and ERs, was used in this study. Individual in-depth semistructured face-to-face interviews were conducted to explore CEs’ experiences and needs while incorporating other stakeholders’ perceptions, which is well served by a naturalistic, qualitative inquiry and triangulation of perspectives [38]. The data from the interviews were subsequently coded for thematic data analysis.

Hence, the study design adopted for this research allows the issues surrounding CFWP in Hong Kong to be explored via the lenses of multiple stakeholders who may harbor diverse and potentially contrasting perspectives. This method is valuable for health science research in developing interventions (ie, CFWPs) because of its flexibility and rigor in studying complex phenomena using several data sources [37].

### Participants

The study participants included three groups: CEs caring for older adults, ERs such as company management personnel, and HPs providing ancillary services for CEs. The following inclusion criteria were used for recruiting CEs: residents of Hong Kong, employed full-time, concurrently taking up a caregiver role for either their parents or spouses aged older than 60 years, able to provide consent, and that participation would not adversely affect their health or well-being. The eligibility criteria for ER were business owners or executives of a company with at least 10 employees and at least 5 years of management experience; for HP, licensed individuals who provided health care services and had at least 5 years of experience related to caregiver service were eligible.

As the time frame for data collection was limited, a purposive sample was used to select participants who already had some CFWPs-related background knowledge or experience. Potential participants were initially identified by referrals from a scholar in a local research institute specializing in caregiver welfare, followed by a review of participants’ prior participation in policy forums and public campaign platforms. This process ensured that the participants were familiarized with the concepts of CE status and the relevant rights and burdens in Hong Kong. Additional participants were identified through the snowballing technique.

### Study Instrument

The semistructured in-depth interviews were conducted using a discussion guide consisting of 17 questions (Multimedia Appendix 1) related to three aspects: (1) respondents’ personal experience with CEs, (2) their attitudes and preferences for policies, and (3) their perceptions of a caregiver-friendly workplace. The discussion guide was developed based on (1) a thorough literature review, (2) the master’s thesis of the first author [39], and (3) the recommendation of two expert panels of researchers specializing in the field of caregivers (ELW from the Chinese University of Hong Kong and XB from the Hong Kong Polytechnic University).

The interview guide was adapted for each participant type. Thus, three sets of interview guides directed the in-depth individual interviews for the three groups of participants. The interview guide for the CEs consisted of a checklist inquiring about the basic demographics of the CEs and their care recipients, followed by open-ended questions about their personal experience on the caregiving journey, encounters on their dual roles, and their interactions with regulatory frames and other actors within the workplace context. The interview guide for ERs started with a checklist to establish basic information about the company, followed by questions about their perspective on the current situation and potential for CFWP. For HPs, the guide outlined their observations and perspectives on CE’s experience and CFWP.

### Interview Process

The initial interviews were conducted face-to-face in informants’ offices but later switched to videoconferencing due to the COVID-19 pandemic. All interviews were audio recorded. Interviews ranged from 20 to 70 minutes. The interviews were conducted in Cantonese (Hong Kong dialect) and then translated and transcribed into English verbatim.

The participant’s written consent and demographic information comprising their age, sex, working position, and marital status were obtained before the interview. During the interview, conversation starters were used to open up the participants so they would feel more at ease and provide the most candid response. Prompts were used to elicit more specific and detailed responses to fully explore their experience and perception [40,41].

### Thematic Framework Analysis

Thematic framework analysis based on the conceptual guiding framework was adopted. Data coding and analysis were independently conducted by two researchers (MML and XB) to ensure the consistency and reliability of the translation from Chinese to English.

The five stages of the analytical process comprised familiarization, identification of a thematic framework, indexing, charting, and mapping and interpretation [42]. The MAXQDA 2018 analysis tool (VERBI GmbH) was used to index all transcribed data. A total of 209 indexes were charted into 4 parent themes and 65 subthemes. The last stage, mapping and interpretation, was used to gauge the prominence of key themes and subthemes across the full list of participants. While the guiding framework informed the specification of questions and the main categories or themes of interest, the themes specified and explicated by this inquiry reflect participants’ views. In other words, any strategy or recommendations emerging from this research echo the participants’ attitudes, beliefs, and values [43]. Data credibility was established by obtaining validation from some participants on the accuracy of how their experiences were registered in the form of interview transcripts. Some participants were also invited to review the interpretations to ensure their beliefs were accurately represented, thereby minimizing bias.

### Ethical Considerations

Research ethics approval was granted by the Imperial College Research Ethics Committee and The Hong Kong Polytechnic University Department of Applied Social Science Research Committee on June 6, 2018 (reference 18IC4581) and May 1, 2018 (reference HSEARS20180413002), respectively. Written consent was obtained from interview informants after the purpose of the study, and the plan for data confidentiality, analysis, storage, and dissemination was explained to them. All data were anonymized and kept in password-protected folders accessible only to the project supervisor and the research student. Participants were not provided monetary or material compensation for participating in this study.

## Results

### Participant Demographics

A total of 15 participants were interviewed between May and June 2018. The CEs (n=9; CE1-9) comprised 4 female and 5 male participants aged 20-70 years from four industries (banking or finance, technology, service, and education), with caregiving experience ranging from 0.5 to 13 years (Table 1). Three of the CEs had previously quit jobs because of caregiving roles. The major conditions leading to the caregiving roles were depression, dementia, physical disabilities, and tumors.

The HPs (n=3; HP10-12) comprised a geriatrician (HP10) who has been engaged in geriatric medicine for over 35 years in private and public practice and two social workers (HP11 and HP12) with over 5 years of experience in different contexts, such as crisis intervention, end-of-life care management, and daily support services (Table 2).

The company personnel (n=3; ER13-15) were drawn from big, medium, and small enterprises engaging in retail, law, and technology, respectively (Table 3). Only ER15 works in a company with a Family-Friendly Employers Scheme by the Hong Kong Government [44].

**Table 1:** Characteristics of CEs^a^ included in this study.

Informant code	Sex	Age group (years)	Industry (seniority)	Condition of the care recipients	Experience as a caregiver (years)	Ever quit a job because of the caregiving role
CE1	F^b^	21-30	Bank (junior)	Brain tumor	5	No
CE2	F	51-60	Retired; bank (senior)	Depression	6	No
CE3	F	21-30	Accounting (middle)	Lung cancer	2	Yes
CE4	F	41-50	Technology	Depression	10	No
CE5	F	31-40	Primary school teacher	Joint disorder	2	No
CE6	M^c^	51-60	Security guard	Physical disabilities	0.5	No
CE7	M	Older than 60	Retired; bank (senior)	Dementia	13	Yes
CE8	M	41-50	Servicing industry (middle)	Dementia	10	No
CE9	M	41-50	Secondary school teacher	Stroke	8	Yes

^a^CE: caregiver-employees.

^b^F: female.

^c^M: male.

**Table 2:** Characteristics of HPs^a^ included in this study.

Informant code	Profession	Years of practice
HP10	Geriatrician	>35
HP11	Social worker and gerontologist; specializes in end-of-life care	>5
HP12	Social worker; specializes in caregiver services	>5

^a^HP: health care professional.

**Table 3:** Characteristics of ERs^a^ included in this study.

Informant code	Company size	Industry	Position	Awardee of family-friendly employers
ER13	200	Legal	Owner	No
ER14	10	Technology	Senior Manager	No
ER15	2000	Retails	Director, Human Resources	Yes

^a^ER: employer.

### Current Overview

Interviews with all three CFWP stakeholder groups indicated the lack of formal workplace policy directly addressing CE issues. CEs further highlighted that any accommodation to their needs was made by management on a case-by-case, informal, and discretionary basis. Only two participants reported some form of formal workplace support for CEs. These family-friendly policies included a home office program and monthly 2-hour early leave for CE1 and CE4. These policies applied to all employees where CE1 and CE4 worked. Both CEs perceived these policies positively, exemplified by the remarks:

It makes a difference. It has reduced stress drastically. It is much easier to manage time, physical health (tiredness), and mental and emotional needs.

CE4

### Thematic Analysis

#### Overview

The thematic analysis yielded 65 codes (Multimedia Appendix 2) categorized into four main themes: (1) lacking leadership, (2) cultural factors, (3) role struggles, and (4) inadequate support.

#### Lacking Leadership

A lack of guidance in laws or social policies for realizing CFWP in Hong Kong was evident during the interviews. All ER participants perceive that companies lack the resources, knowledge, and experience to support CEs. They consider government leadership essential, such as subsidies, guiding policy, and technical support. All three ER informants expressed willingness to adopt CFWP if the government takes the lead first. For example, one ER stated:

Government taking the lead is important. As long as there are some initiatives in the Employment Ordinance, corporates will initiate to follow...Citing paternal leave is a good example...How can he be part of the family throughout the journey? If considered, you could be surprised what the corporates would offer.

ER15

Within the organizational leadership, many operational factors and competing priorities influence ERs’ decisions in adopting CFWP in Hong Kong, such as cost, fairness, potential uptake, and inadequate resources. ER13 reflected that caregiving roles and mental illnesses are stigmatized in Hong Kong, limiting potential service utilization. Smaller companies are hesitant to adopt CFWP officially for the potential cost implications and further responsibilities it might entail. ER14 remarked that formalization may unleash other obligations. ER14 and ER15 admitted they have other priorities, such as childcare, over CFWP. For example, ER15 highlighted, “Corporates might perceive not all older adults need help, but all children need care.” HP10 confirmed that children-friendly work arrangements are common but not CFWP in Hong Kong.

#### Cultural Factors

CFWP aims to promote holistic integration of work and life by enabling CEs to balance their roles as employees and caregivers. However, some observations made by participants seem to suggest that the fundamental tenets of CFWP may be at odds with cultural and workplace values in the Hong Kong setting. The concept of work-life separation, rather than work-life balance, is highly prevalent in Hong Kong. A total of 6 (67%) out of 9 CEs indicated that it is an established norm in Hong Kong to separate personal or private concerns from work, if not altogether, rendering them secondary to work concerns. In other words, personal issues should not be imposed on workplace*s.* Failure to detach from personal stress at work could entail a reproach from management. For instance, CE7 recalled that his manager remarked, “Don’t bring this burden to work. Once you clock in, you better not have this burden in your mind.”

Four CE participants stated that the prevalent corporate attitude in Hong Kong prioritizes business interests over employees’ well-being. The participants highlighted a culture of assumption of total work commitment from employees that leaves little room for considering CEs’ concerns. They further intimated that this culture makes it difficult for CEs to raise the issue of caring for loved ones with their managers. HP10 shared this sentiment rather strongly while stating, “Employers expect slavery, working 10 hours, better not to go home, not taking leave, not getting sick, without seniors at home, no marriage, no relationship, and no children.”

This attitude of management caused acute stress for employees and potential conflict with management when urgent health issues of the family care recipients need to be dealt with while the CE is at work. At these times, CEs find themselves in a dilemma of multiple roles and uncertainties. For example, one CE stated:

It was so stressful every time my mother called when I was at work...I was so nervous. Because of the long-term mental stress, I have hypertension.

CE7

All CEs lamented a general lack of compassion from their ERs and colleagues. CEs must take formal leave because “caregiving roles occur outside work hours.” As a result of the unsympathetic attitude, 6 CEs chose not to disclose their CE status and remained silent, while others became more stressed or decided to quit. CE5 described her organization’s management as so apathetic that she chose not to disclose her chronic condition as well. She regarded her role in the organization as “replaceable.” Therefore, letting management know her caregiving role was not an option. She felt that she had no power or involvement in the decision-making process concerning her disease or her caregiving role because of the hierarchy of her organization. CE5 and HP11 attributed this passivity from the ERs to the assumption that the government should take full responsibility for supporting CEs.

CEs also spoke of colleagues’ indifference to their circumstances. Fear of gossip limits CEs from disclosing their circumstances. Disclosing could be perceived as an excuse to do less work, as exemplified by a statement:

Trust is important, so information about me will not be spread around...I don’t want people to see it as an excuse [weakness]. I don’t want people to see me using my mother’s condition as an excuse to do less work.

CE3

CE6, a security guard, believes that disclosure could dangerously affect his position in the organization. Conversely, CE4, a senior manager, expressed no hesitation or concerns regarding disclosure of his CE status. This indicates that seniority in the corporate hierarchy influences how empowered CEs may feel about disclosing their status.

The notion of the primacy of work over personal concerns is also reflected in CEs’ behavior. Most CEs took it upon themselves to overcome additional burdens at work and were either not keen or could not seek help and share burdens with others. They expressed high expectations of themselves as employees and caregivers, compounding the stress. One HP noted:

They (CEs) will just impose the problems on themselves. They choose not to take absences but work until midnight to care for the older adults.

HP10

This work ethic exacerbates the stress experienced by the CEs.

#### Role Struggle

All CEs expressed experiencing struggles navigating the different roles as employees, caregivers, parents, partners, and friends. They felt the urge to reprioritize values in life as they often take on a reverse role, from a child to a guardian for their frail parents. CEs reported that the caregiving role influenced their behaviors and decisions, like spending patterns or quitting a job*.* For instance, one CE stated:

I didn’t want to abuse the system or the job and wanted to prioritize my students’ learning experience, and that’s why I decided to quit.

CE9, teacher

CEs sacrificed their time to undertake the caregiver role at the expense of self-care, potentially causing a loss of personal identity and difficulties connecting with themselves and others. As CE1 stressed, “The biggest change is the loss of friends and my personal time. All my time was devoted to family needs.” In the case of CE7, he was abandoned by his wife after his full-time caregiving role forced him to quit his job. Thus, reprioritization of values could wreak havoc on family integrity. In addition, reprioritization may result in unmet psychological and emotional needs, introducing an identity struggle that further overwhelms the CEs. In this regard, CE7 remarked, “I need to look at myself as another person to live up to my caregiving role.”

It is difficult to strike a balance between caregiver and employee roles. The intensity and stress of these roles are inseparable and nonexclusive, which may lead to a downward spiral if no support is available. New CEs may be more prone to this balancing problem because they lack institutional support and resources to prepare for these dual roles. For instance*,* one CE stated:

It was very difficult to adjust at the beginning...Letting go of work stress and switching to caregiver mode was very difficult.

CE1

Conversely, work could also be a protective factor for CEs to take on caregiving roles. Engagement at work can alleviate the caregivers’ stress if balance can be attained. This was best exemplified by the statement:

If the caregiving role is 24/7, you will lose the meaning and make caregiving an obligation. It will become a burden. Especially in Hong Kong, where living space is so small. If you only take on the caregiving role, it can feel like being trapped in a cage.

CE8

The unavoidable familial tension—reported as “unresolved childhood emotions”—further rarifies the role struggle. HP12 spoke about how the inner child of the caregivers can be a vulnerability: “Since the care recipients are the parents who have shaped a large part of the caregivers’ lives, a single gesture or action could trigger emotional stress from trauma as far [back] as those from childhood.” CE5 exemplified inner child struggle as well. She reflected that she found it unfair that she had to care for her father, who had done little to support the family during her childhood. Even though she has become financially independent, CE5 would readily associate the overwhelming stress of the caregiving role with the powerlessness and lack of confidence in her child-self because of the wounds caused by her father. These emotions could intensify the present role struggle.

#### Inadequate Support

CEs exhibited a feeling of hopelessness mainly because of the uncertainties they faced. This hopelessness is exacerbated by the minimal assistance and support received, as explained by one HP:

Hopelessness...because chronic illnesses have no foreseeable end. The uncertainty of knowing how long the suffering will last causes hopelessness. The caregivers’ task lists don’t end either. It never ends. It is endless.

HP11

There is limited information to prepare and enable caregivers to live up to their roles. Besides limited information from doctors, there is a lack of reader-friendly, timely, and high-quality information. HP 10 remarked, “There is a serious need for information support...Hearsay is commonly found but useless.” All CEs were frustrated in finding the quality information they needed to live up to their roles, including those related to social welfare support and handling the needs of care recipients. Most CEs had little idea about available social welfare support resources because of limited public awareness and promotion programs. In this context, one CE stated:

The hospital didn’t actively promote them to us. And we didn’t expect there is this kind of thing. It was difficult to find appropriate information. Doctors at public hospitals would not have the time to explain—only standard treatment procedures. But they didn’t talk about the side effects or things about daily care to know. I was quite lost.

CE3

Most CEs perceived the information provided by social welfare support as inadequate. One of the social worker informants confirmed that due to potential conflicts of interest in recommending a specific service provider to caregivers, it is normal for caregivers to receive a long list of providers without recommendations, which can be confusing. The inadequate quality of information and the lack of promotional programs cause an information gap that makes CEs’ caregiving journey challenging.

Accessing social welfare support is limited because of the high qualification threshold. Most cases are in the middle of the scale and ineligible for financial or physical support like respite care or home care services. In addition, the resources designated for caregivers are generally meager. One CE responded:

The social worker has only 1% of her work duties devoted to handling cases...she made decisions on my behalf without telling me about the rationale and the context.

CE9

Such negative experiences further discourage CEs from seeking social welfare support. ERs noted the need to match the services of providers with users, particular conditions they are facing, and the stage in the caring process. HP13 reported that CEs’ characteristics influence service quality and satisfaction. Matching is crucial in the first stage to ensure good quality, according to ER9.

Health care providers agreed that social welfare support is inadequate because of fragmentation and lack of crisis intervention. Crisis intervention services are available only to hospitalized cases. The chances of accessing them after discharge are minimal. The care system is fragmented and superficial because social workers have other challenging roles besides carer services. Social workers might chase the number of cases under their purview instead of going deeper individually by enhancing the caregivers’ well-being or counseling them.

Besides limited resources and services, operations and priority matrix do not favor caregivers’ referrals for social support, as exemplified by the following statement by HP10: “Relying exclusively on their doctors is not enough when these doctors are extremely busy.” CE3 further reflected that health care providers’ attitudes often lacked empathy and professionalism, resulting in disappointment and frustration. The lack of empathy or indifferent attitude of health care practitioners intensifies CEs’ powerlessness. In this regard, CE3 stated, “Since time and resources are scarce in the public hospital, they wouldn’t prepare you mentally for the situation. They would just tell me the symptoms and options and seek our consent for surgery immediately. There was no time allowed for our consideration. Everything was mechanical.”

There are also substantial barriers to accessing professional mental health services for caregivers. For example, waiting time is considerably long, and according to HP12, “only serious cases, such as suicidal attempts, might be considered for the clinical mental health services.”

## Discussion

### Principal Findings

This study explored the CFWP-related views and experiences of multiple stakeholders (CEs, ERs, and HPs) in Hong Kong, guided by a theoretical framework that oriented the interest toward exploring CE experiences in depth (IVT and spillover theory) and exploring the potentials of CWFP as new ideas within the broader context (PARIHS framework). Principal findings were broadly organized under two context-related (“lacking leadership” and “unfavorable culture”) and two facilitation-related (“role struggle” and “inadequate support”) themes.

Regarding CFWP, Hong Kong lags behind other aging Asian societies, such as Japan and Taiwan, which have already adopted 93 [45] and 21 [46] leave days for CEs, respectively. Lack of clear leadership and confusion in public policy roles and guidance was highlighted as one of the primary reasons why explicit CFWPs are absent in Hong Kong, despite the urgent need for CEs and the apparent willingness of ERs to adopt CFWP. The government taking the lead in establishing guiding frameworks for CFWP, both in law and administration, appears to be the critical missing link. Given that Hong Kong’s health care system bears colonial inheritance [47], the United Kingdom’s Care Act could prove instructional. The UK Care Act was implemented in 2014 to protect the rights of caregivers. The law provides provisions for the local authorities to identify CEs’ needs by assessing private companies [48]. These provisions allow the UK government to strategically partner with private companies and provide support and services for ERs to achieve caregiver-friendly working environments [49]. Similar legal protection and policies adapted to the local context will be instrumental in widespread CFWP adoption among Hong Kong companies.

Regarding organizational leadership, welfare ideology and operational concerns are the two factors hindering the formulation and uptake of CFWP. Hong Kong’s long-standing universal welfare ideology, described as distorted toward the government presumably providing for welfare in aging and antipoverty programs [50], fuels companies’ denial of their corporate responsibility in addressing the needs of the CEs. This study also shows that operational factors, such as cost, potential uptake, and inadequate resources, prevent CFWP from gaining prominence in the organizational leadership agenda. However, the absence of CFWP is far more costly in the long run due to increased absenteeism, reduced work productivity, increased turnover, and work disruptions [51]. Sustainable long-term organizational growth would require well-considered organizational leadership that addresses this substantive issue.

In addition, local cultural factors were also identified as hindrances to CFWP adoption, such as issues concerning work-life separation, total work commitment, and lack of compassion from management. The Confucian work ethic of hard work, perseverance, and patience is also deeply embedded among Hong Kongers [52]. The sentiments and behavior reflected in our study affirm the prevalence of these values and further indicate that these may not always be compatible with the underlying principles of CFWP. The associated taboos and risks of speaking up, as perceived and experienced by CEs, further demonstrate the assertions of IVT that explain the risks and inappropriateness of speaking up.

Older adults may have diverse and unexpected health care needs that are not always associated with chronological age [53]. Thus, adopting a caregiver-friendly workplace culture conducive to compassion and acceptance of CE’s circumstances is necessary. Such cultures include supportive management, a trusting environment, and establishing top-to-bottom leadership [54]. An empathic and caregiver-friendly culture would potentially encourage CEs to identify themselves (rather than implicit voice spaces), and incorporating flexibility and acceptance into business management and work culture enhances employee commitment [55].

Regarding role struggles, our findings indicate that there may be positive and negative spillovers between work and family, upholding the constructs of spillover theory. For some CEs, work can serve as a break from caregiving roles, and hence, be a protective factor, while the high burden of dual roles can force some CEs to quit work, sacrifice self-care, or even get abandoned by family members. For instance, a study focusing on the well-being of Hong Kong male caregivers reported that work has protective effects by improving caregivers’ resilience and overall well-being [56]. Therefore, role-balancing is key because it rewards CEs with a sense of satisfaction and fulfillment and also avoids any ramifications by giving up paid employment for caregiving roles [57]. CFWP, which promotes and engages CEs to balance work and life, is critical for positively managing this spillover effect. These findings are consistent with existing literature that CFWP reduces absenteeism and sick leave rates and increases employees’ productivity, loyalty, engagement, and morale [58-60].

The inadequacy of support was amply reflected by CEs and HPs alike. Even though family caregiving has become more demanding, complicated, and longer-lasting than in the past, caregivers are often underprepared [61]. This study further highlights that CPWP is most needed by CEs who have just commenced the caregiving role and experience acute crisis because of little preparation and knowledge about their new role. This, in turn, emphasizes how support for new caregivers is critical for the short and long-term well-being of both CEs and their care recipients. Caregiver preparation and education are vital in reducing psychological stress and coping with the situation better [62-64]. Companies can bridge the information gap and facilitate matching according to their unique demographics and the needs of caregivers. CFWP is, therefore, imperative to help new CEs navigate their caregiving journey.

Previously, caregiving tasks in Hong Kong could be shared among siblings when family sizes were larger. However, nuclear families have become the norm in Hong Kong, with an average household size of 2.8 members [65]. Future generations are expected to have even fewer siblings sharing caregiving responsibilities, which is bound to compound and intensify the burden and sense of powerlessness. Therefore, effective CFWP is needed to ensure support for income earners to avoid the myriad adverse effects on individuals, society, and businesses.

### Limitations

The major limitation of this study is that we predominantly recruited CEs engaged in full-time work, which may not fully reflect the views of CEs who are part-time or unemployed. In addition, CE participants in this study were well primed to reflect on these issues as they were already actively engaged in policy consultations for CFWP, limiting the generalizability of our findings.

### Conclusions

This study is the first inquiry into the experiences of CEs and factors that may influence the adoption of CFWP in Hong Kong. The qualitative methodology and the purposively identified participants have provided a glimpse into the lives and tribulations of this otherwise invisible but growing population group. Our findings strongly indicate that Hong Kong’s current workplace policy frameworks fall short of meeting the immediate needs of CEs and the long-term interests of companies and society. We urge actors to explore and adopt potential CWFP for Hong Kong, considering the complexity of factors explored in this study, including unique cultural dynamics and structural factors related to the aging population and ever-increasing health care burden. Future research should identify CEs who need the most support and pinning down the most optimal forms of regional law and organizational policy.

## Appendix

Multimedia Appendix 1 The interview guide.

Multimedia Appendix 2 Emergent themes, subthemes, and codes from thematic framework analysis.

## Abbreviations

CE: caregiver-employee
CFWP: caregiver-friendly workplace policy
ER: employer
HP: health care professional
IVT: implicit voice theory
PARIHS: Promoting Action on Research Implementation in Health Services

## Data Availability

The datasets generated during and/or analyzed during this study are not publicly available for privacy reasons but are available from the corresponding author upon reasonable request.
