# Supplementary material for: Perceptions and Experiences of Caregiver-Employees, Employers, and Health Care Professionals With Caregiver-Friendly Workplace Policy in Hong Kong: Thematic Analysis
Source: Interact J Med Res. 2025 Feb 10;14:e58528. doi: 10.2196/58528 (PMC11851026; doi:10.2196/58528)
Supplement: Multimedia Appendix 1 [file ijmr_v14i1e58528_app1.docx]

**Fact sheet about the informant:**

1. Position
2. Age
3. Gender
4. Marital status and number of children (age)

**Questions Guide (17 questions)**

1. **Personal experience**
2. Are you aware of caregiver-employees in your company or career?
3. Are you aware of CFWP?

If so,

- - - - 1. How?
        2. When?
        3. Any comment in the context of Hong Kong?

1. Are you aware of their needs?
   1. From your personal experience
   2. From your colleagues
2. Imagine what will happen after 50 years: the dependency ratio of Hong Kong will drop from 4.18 to 1.18 per elderly, with longer life expectancy; how would you see this become?
3. How would you respond if a staff member asks to take a leave to care for an older person who just got sick?
4. **Attitude and policy preference**
5. Do you think CFWP is necessary for Hong Kong?
6. Would you consider it difficult or as easy as implementing maternity/ paternity leave?
7. If public needs, even though it might contradict the business objective, how would you see it?
8. Do you think CFWP will benefit talent retention and/or attracting talent?
9. Ideally, how would you want CFWP to be?
   1. In law
   2. In policy
10. And ideally, how would you want it to be in terms of government support in promoting CFWP?
11. **Caregiver-friendly working environment**
12. Do you think giving emotional support is necessary?
    1. If yes, how do you think it is done?
    2. If not, why?
13. How would you address the following barriers to improve CEs' experience and well-being at work?
    1. Business interest domination
    2. Uncompassionate views from management and colleague
    3. Confucian work ethics
    4. Work-separation
14. Creatively speaking, how would you change your company corporate to create a more caregiver-friendly workplace environment?
15. Please rate the following from your point of view – which will help the caregivers the

|  |  | 0 least important -10 most important |
| --- | --- | --- |
| 1. | Caregiver-inclusive corporate culture |  |
| 2. | Paid caregiver leave ( not counting maternity leave and maternity leave ) |  |
| 3. | Unpaid caregiver leave |  |
| 4. | Bereavement leave |  |
| 5. | Flexible working hours |  |
| 6. | Flexible work locations |  |
| 7. | Switch to a part-time mode |  |
| 8. | Unpaid leave |  |
| 9. | Aiding medical needs/insurance of employees' parents ;  Please specify the insured as: ______ |  |
| 10. | Information / Carer Skills / Guide to Community Care Resources |  |

1. Have you thought of other policies that can help you complete the tasks of a caregiver? _________

**Closing**

1. Is there anything we have not discussed about your experience that you want to bring up now?
