# Supplementary material for: Perceptions and Experiences of Caregiver-Employees, Employers, and Health Care Professionals With Caregiver-Friendly Workplace Policy in Hong Kong: Thematic Analysis
Source: Interact J Med Res. 2025 Feb 10;14:e58528. doi: 10.2196/58528 (PMC11851026; doi:10.2196/58528)
Supplement: Multimedia Appendix 2 [file ijmr_v14i1e58528_app2.docx]

| **Color** | **Parent code** | **Code** | **Coded segments of all documents** |
| --- | --- | --- | --- |
| 1 |  | Management | 10 |
| 2 | Uncompassionate | Colleagues | 5 |
| 3 | Sense of powerlessness | Poor public health services for both CEs and the elderly | 5 |
| 4 | Sense of powerlessness | Inadequate social welfare support | 4 |
| 5 | Sense of powerlessness | Lack information | 6 |
| 6 | Out of balance | Good balance | 1 |
| 7 | Out of balance | Out of balance | 3 |
| 8 | Inadequate social welfare support | Patient-centered | 2 |
| 9 | Identity struggle | Loss of personal time | 5 |
| 10 | Identity struggle | Reprioritize values in life | 5 |
| 11 | Identity struggle | Loss of personal identity | 1 |
| 12 | Identity struggle | Emotions & reconciliation | 6 |
| 13 | Hong Kong Style | Business interest domination | 4 |
| 14 | Hong Kong Style | Work-life separation | 6 |
| 15 | Hong Kong Style | Confucian work ethics | 7 |
| 16 | Helpful Policy | CE identification | 3 |
| 17 | Helpful Policy | No-paid leave | 1 |
| 18 | Helpful Policy | Two-hours early leave | 1 |
| 19 | Helpful Policy | Matching the CEs and service providers | 2 |
| 20 | Helpful Policy | Counselling | 1 |
| 21 | Helpful Policy | Time off | 1 |
| 22 | Helpful Policy | Preparing caregivers through education | 2 |
| 23 | Helpful Policy | Home office | 2 |
| 24 | Helpful Policy | Part-time role | 1 |
| 25 | Helpful Policy | Elder Care Resources and Referral | 2 |
| 26 | Helpful Policy | Support group | 1 |
| 27 | Feelings | Guilt | 2 |
| 28 | Feelings | Stress as an employee | 1 |
| 29 | Feelings | Worries as a caregiver | 2 |
| 30 | Feelings | Stress as a caregiver | 1 |
| 31 | Feelings | Fears | 7 |
| 32 | Feelings | Dangerous | 1 |
| 33 | The elephant in the room | Aware of the CEs' needs | 5 |
| 34 | The elephant in the room | Informal Policy | 9 |
| 35 | Cultural difference | Hong Kong style | 10 |
| 36 | Consequences of no CFWP | To corporates | 2 |
| 37 | Consequences of no CFWP | To society | 0 |
| 38 |  | Caregiver-specific support | 7 |
| 39 |  | Performance of CE | 4 |
| 40 |  | Philosophy of being family-friendly | 3 |
| 41 |  | Need for a cultural paradigm shift | 2 |
| 42 |  | Trust | 2 |
| 43 |  | Gender | 1 |
| 44 |  | Concerns of ERs | 6 |
| 45 |  | ER's other priority | 4 |
| 46 |  | Perception of CFWP | 3 |
| 47 |  | Benefits to ERs | 5 |
| 48 |  | Cultural difference | 0 |
| 49 |  | Identity struggle | 0 |
| 50 |  | Sense of powerlessness | 2 |
| 51 |  | Uncompassionate | 2 |
| 52 |  | The elephant in the room | 1 |
| 53 |  | Out of balance | 0 |
| 54 |  | Learning curve | 4 |
| 55 |  | Outsource | 5 |
| 56 |  | Double patients | 1 |
| 57 |  | Family support | 5 |
| 58 |  | Time Conflict | 6 |
| 59 |  | The stigma of getting help | 3 |
| 60 |  | Silent | 3 |
| 61 |  | Newly minted CE | 4 |
| 62 |  | Consequences of no CFWP | 4 |
| 63 |  | Absence of guiding framework | 4 |
| 64 |  | Helpful policy | 1 |
| 65 |  | Feelings | 0 |
| ***Total*** | | | ***209*** |
